# *Fusobacterium nucleatum* induces invasive growth and angiogenic responses in malignant oral keratinocytes that are cell line- and bacterial strain-specific

**DOI:** 10.3389/fcimb.2024.1417946

**Published:** 2024-09-02

**Authors:** Ajith Selvaraj, Gavin McManus, Claire M. Healy, Gary P. Moran

**Affiliations:** ^1^ Division of Oral Biosciences, Dublin Dental University Hospital and School of Dental Science, Trinity College Dublin, Dublin, Ireland; ^2^ School of Biochemistry and Immunology, Trinity Biomedical Sciences Institute, Trinity College Dublin, Dublin, Ireland; ^3^ Division of Oral and Maxillofacial Surgery, Oral Medicine and Oral Pathology, Dublin Dental University Hospital and School of Dental Science, Trinity College Dublin, Dublin, Ireland

**Keywords:** *Fusobacterium nucleatum*, oral cancer, invasion, angiogenesis, inflammation

## Abstract

*Fusobacterium nucleatum* is an anaerobic commensal of the oral cavity recently reported to be associated with cancers of the gastrointestinal tract and oral squamous cell carcinoma (OSCC). In this study, we investigate the impact on oral keratinocytes of infection with a genetically diverse set of strains of *F. nucleatum* subsp. *polymorphum* recovered from patients with oral dysplasia (n=6). We employed H357 oral keratinocytes derived from a stage 1 OSCC and H376 cells derived from a stage 3 OSCC. Adhesion phenotypes were strain specific, with 3/6 clinical isolates examined exhibiting higher adherence to the stage 3 H376 cell line. Conversely, intracellular invasion was greatest in the H357 cells and was associated with specific transcriptional responses including autophagy and keratinization. Infection of both H357 and H376 cell lines induced transcriptional and cytokine responses linked to cancer cell migration and angiogenesis. *F. nucleatum* infection induced greater levels of MMP9 secretion in the H376 cell line which was associated with enhanced motility and invasion phenotypes. Additionally, the degree of *F. nucleatum* induced invasive growth by H376 cells varied between different clinical isolates of *F. nucleatum* subsp. *polymorphum.* Blockage of CCL5 signalling using the inhibitor metCCL5 resulted in reduced keratinocyte invasion*. F. nucleatum* infection also induced expression of the pro-angiogenic chemokine MCP-1 and the angiogenic growth factor VEGF-A resulting in increased capillary-like tube formation in HUVEC cells, most significantly in H376 cells. Treatment of HUVEC cells with resveratrol, a VEGF-A signalling inhibitor, significantly attenuated *F. nucleatum* induced tube formation. Our data indicate that the outcomes of *F. nucleatum-*oral cell interactions can vary greatly depending on the bacterial genotype and the malignant phenotype of the host cell.

## Introduction

*Fusobacterium* species are Gram-negative obligate anaerobes belonging to the phylum Fusobacteria (Han, 2015; Brennan and Garrett, 2019). There are four subspecies of *F. nucleatum*, namely *animalis, nucleatum, polymorphum* and *vincentii*, each of which have been shown to exhibit different adhesive and biofilm forming capabilities (Gharbia et al., 1990; Muchova et al., 2022). Within the last decade, *F. nucleatum* has come under increased scrutiny due to its association with cancers of the gastrointestinal tract, particularly colorectal carcinoma (CRC) (Castellarin et al., 2012; Kostic et al., 2012). The presence of *F. nucleatum* within CRC tissue has been associated with increased tumor staging, chemoresistance and poor patient outcomes (Mima et al., 2016; Yu et al., 2017; Kunzmann et al., 2019). Recent research has suggested that *F. nucleatum* promotes a proinflammatory microenvironment in CRC that promotes cell proliferation, cell migration and metastases (Rubinstein et al., 2013, 2019; Coppenhagen-Glazer et al., 2015; Gur et al., 2015; Umana et al., 2019). In CRC, *F. nucleatum* has been proposed to modulate cellular properties due to the interaction of two well characterised adhesins, namely Fap2 and FadA with host cells (Gur et al., 2015, 2019; Bullman et al., 2017; Casasanta et al., 2020; Niño et al., 2022). The Fap2 adhesin targets D-galactose-β(1–3)-*N*-acetyl-D-galactosamine (Gal-GalNAc) residues which are upregulated in CRC tissues and in breast tumors and this enhanced presentation of Gal-GalNAc has been suggested as the reason for enrichment of *F. nucleatum* in these tumors (Abed et al., 2016; Parhi et al., 2020). Deletion of Fap2 in *F. nucleatum* has shown that this protein is required for IL-8 and CXCL1 induction which are involved in host cell migration phenotypes (Casasanta et al., 2020). Additionally, the FadA adhesin has been demonstrated to bind to E-cadherin on host epithelial cells and has been reported to activate β-catenin signalling and activation of pro-oncogenic and inflammatory responses (Rubinstein et al., 2013, 2019).

The role of *F. nucleatum* in the progression of oral squamous cell carcinoma is less well defined. *F. nucleatum* has been identified as a major component of the intratumoral microbiome in OSCC where it has been shown to be metabolically active (Yost et al., 2018). Using FISH, Galeano Niño et al. showed that *F. nucleatum* can be found in localised micro-niches within OSCC tissues and that these areas exhibited localised immunosuppressive effects including increased expression of ARG1 (arginase 1) the T-cell-inhibitory receptor PD-1 and decreased expression of wild-type p53 (Niño et al., 2022). Clinically, it has been reported that the abundance of *Fusobacterium* present in the mouth increases with tumor staging, from 4.35% in stage 1 OSCC to 7.92% in stage 4 OSCC (Yang et al., 2018).

Infection of oral malignant cell lines with *F. nucleatum in vitro* was shown to induce epithelial-mesenchymal transition (EMT), oncogene expression and promote invasive growth (Harrandah et al., 2020; Shao et al., 2021). *F. nucleatum* has also been shown to accelerate carcinogenesis in chemically induced OSCC murine models (Gallimidi et al., 2015; Harrandah et al., 2020). We have recently shown that oral mucosal surfaces, including dysplastic tissue and OSCC are primarily colonised by *F. nucleatum* subsp. *polymorphum* and that these isolates exhibit a high degree of genetic variation, including a high degree of variability in the copy number of fadA- and fap2-related adhesin encoding genes (Crowley et al., 2024). In this study, we attempt to determine if these clinically relevant and genetically diverse strains of *F. nucleatum* subsp. *polymorphum* have different interactions with host cells. In addition, we also examine the impact of host cell phenotype on this interaction using two different OSCC cell lines, namely a stage 1 tumor derived cell line (H357) and a stage 3 derived cell line (H376). Our data indicate that the outcomes of *F. nucleatum-*host cell interactions can vary greatly depending on the bacterial genotype and the nature of the host cell.

## Materials and methods

### Microbial growth conditions

*F. nucleatum* was routinely cultured on BHI agar supplemented with 5% defibrinated horse blood (TCS Biosciences Ltd, UK). Cultures were maintained in anaerobic jars supplemented with AnaeroGen 3.5L sachets (Thermo Fisher Scientific, UK) in 37˚C incubators. For isolation of *F. nucleatum* from clinical swab samples, swabs were cultured on Fastidious Anaerobe Agar (FAA) (Neogen, Lansing, MI, USA), supplemented with 5% (vol/vol) defibrinated horse blood and the antibiotics josamycin (3 µg/mL), vancomycin (4 µg/mL) and norfloxacin (1 µg/mL) [JVN medium; (Brazier et al., 1991)]. For liquid culture, *F. nucleatum* was grown in BHI broth (Sigma-Aldrich, St. Louis, USA) supplemented with 2.5 g yeast extract, 0.3 g L-Cysteine, 5 µg/ml haemin and 1 µg/ml Menadione (Sigma-Aldrich). Overnight broth cultures of *F. nucleatum* were grown to exponential phase, centrifuged (4000 rpm for 5 minutes) and washed three times with sterile PBS before standardising the bacterial suspension at OD_600_ of 0.7 which was diluted in PBS to yield a suspension of 1x10^7^ cfu/ml.

The type strain *Fusobacterium nucleatum* subsp*. polymorphum* NCTC10562 (equivalent to ATCC 10953) was obtained from the National Collection of Type Cultures (NCTC, Porton Down, UK). All clinical isolates of *F. nucleatum* used in this study were obtained from mucosal swabs recovered with ethical approval granted by the Joint Hospitals Research Ethics Committee (JREC; reference no. 2017-11-Chairman’s Actions-7). *F. nucleatum* was recovered from patients with oral epithelial dysplasia attending the Dublin Dental University Hospital by gentle swabbing of the affected mucosal location (see **Table 1**) followed by anaerobic culture on JVN medium as described (Crowley et al., 2024). Isolates used in the analysis (**Table 1**) included strains from mild (60A2), moderate (40A2) and severe dysplasia (41A, 43A3) as well as isolates recovered from contralateral healthy mucosa in the same patients (41B2, 43B1). These strains were subjected to whole genome sequencing (Crowley et al., 2024) and the estimated copy numbers of the major *F. nucleatum* adhesins encoded in each strain are detailed in **Table 1**. *Escherichia coli* strain DH5α was obtained from the Dublin Dental University Hospital microbiological strain collection.

**Table 1:** Details of *F. nucleatum* subsp. *polymorphum* isolates used in this study including copy number of major adhesins from whole genome sequences.

					Adhesin Copy Number**								
Strain	Patient ID	Nationality	Location	Disease*	Fap2	CmpA	RadD	Aim1	FN868	FN1003	FadA1	FadA2	FadA3
NCTC10562	NA	USA	Inflamed gingiva	Inflamed gingiva	2	0	1	2	0	0	1	1	2
40A2	40	Irish	Ventral Tongue	OLK (Moderate dysplasia)	1	0	0	0	0	0	1	0	3
41A	41	Irish	Ventral Tongue	OLK (Severe dysplasia)	2	1	0	0	0	0	1	0	2
41B2	41	Irish	Lateral Border Tongue	Healthy mucosa	2	1	0	0	0	0	1	0	2
43A3	43	Irish	Floor of Mouth	OLK (Severe dysplasia)	2	1	0	0	0	1	1	0	1
43B1	43	Irish	Lateral Border Tongue	Healthy mucosa	2	1	0	0	0	1	1	0	1
60A2	60	Irish	Floor of Mouth	OLK (Mild Dysplasia)	2	0	0	0	0	0	1	0	4

*OLK=Oral Leukoplakia

**Estimated number of putative adhesin encoding genes in each genome identified by whole genome sequencing. Green = no copies of the gene; Orange = 1 to 4 copies as indicated. Data from Crowley et al. (Crowley et al., 2024).

### Cell lines and cell culture

Two malignant oral keratinocyte cell lines were used in this study; H357 cells originated from a SCC of the tongue (stage 1) and H376 cells originated from a SCC of the floor of mouth (stage 3) (Prime et al., 2005). Both malignant cell lines were a gift of Dr. Simon Whawell (Dental School, Plymouth, UK) and were grown and maintained in 75 cm^2^ tissue culture flasks with vented caps (Corning, NY, USA) in Keratinocyte Growth Media (KGM) consisting of Dulbecco’s Modified Eagle Medium (DMEM) containing 2 mM L-glutamine (Gibco, Thermo Fisher Scientific, UK), 10% foetal bovine serum (Thermo Fisher Scientific), 2.6 g/l F-12 Ham nutrient mixture (Sigma-Aldrich), 0.5 µg/ml hydrocortisone (Sigma Aldrich), 10 ng/ml cholera toxin (Thermo Fisher Scientific), 5 µg/ml insulin (Roche Life Sciences, Switzerland), 10 ng/ml epidermal growth factor (R&D Systems, MN, USA), 25 ng/ml adenine, 200 IU/ml penicillin and 200 µg/ml streptomycin (Sigma-Aldrich). Media was replaced every third day and cells were harvested every 5-6 days after reaching 80% confluence and sub-cultured in fresh sterile tissue culture flasks.

TERT-1/OKF6 telomerase immortalized cells (Dickson et al., 2000) were a gift from Dr Antonio Amelio (Department of Oral and Craniofacial health Science, University of North Carolina at Chapel Hill, USA). TERT-1/OKF6 cells (Dickson et al., 2000) were grown using keratinocyte serum free media (SFM) including 5 ng/ml epidermal growth factor and 50 µg/ml bovine pituitary extract (Gibco Thermo Fisher Scientific, UK) supplemented with 200 IU/ml penicillin, 200 µg/ml streptomycin and 0.3 mM CaCl_2_ (Sigma Aldrich). HUVEC cells were cultured in MCDB medium (Thermo Fisher Scientific) supplemented with 8 mM L-glutamine (Thermo Fisher Scientific), 1 mg/ml hydrocortisone (Thermo Fisher Scientific), 10 µg/ml epidermal growth factor (R&D Systems), 200 IU/ml penicillin, 200 µg/ml streptomycin (Sigma Aldrich) and 15% heat inactivated foetal calf serum (Thermo Fisher Scientific).

Cell culture supernatants recovered following *F. nucleatum* infection (MOI 10:1 in DMEM unless stated otherwise) are referred to henceforth as conditioned media (CM) and were used in ELISA and transwell invasion assays. ELISA assays for CCL5/RANTES and CCL2/MCP-1 were performed using Quantikine sandwich ELISA kits (R&D Systems) according to the manufacturer’s instructions. Multiplex analyte assays were performed by Eve Technologies Ltd. (AB, Canada) using the Human Cytokine/Chemokine Discovery Assay (HD48A) and the Human MMP and TIMP 13-Plex Discovery Assay (HMMP/TIMP-C,O). Cytokine assays were performed with a minimum of 3 biological replicates.

### Fluorescent staining

Coverslips coated with epithelial cells were fixed with 3.7% paraformaldehyde, then washed 3 times in 1 ml PBS for 3 minutes. Cells were then permeabilized in 1 ml 0.1% triton X100 buffer for 5 minutes then washed 3 times in 1 ml PBS for 3 minutes. The cells were then treated with a primary anti-E-cadherin antibody (polyclonal goat IgG) followed by a secondary antibody (donkey anti-goat Ig). The cells were then treated with DAPI to stain the nuclei and mounted on glass slides using Mowiol (Sigma-Aldrich) and images were captured using a Leica SP8 Scanning Confocal microscope. To detect the presence of Gal-GalNAc residues on cell surfaces, cells were fixed using 3.7% paraformaldehyde, washed with PBS and incubated with 50 μl of FITC-labelled peanut agglutinin (PNA, Sigma-Aldrich) at a concentration of 100 μg/ml for 30 min and mounted on glass slides using Mowiol (Sigma-Aldrich).

### Bacterial adhesion and invasion assays

For adhesion assays, H357 and H376 cells were grown in 6-well plates to 80% confluency (~1 x 10^6^ cells). Cells were infected with *F. nucleatum* subsp. *polymorphum* (MOI 10:1) in the absence of antibiotics. The plate was spun at 800 x g for five minutes and incubated with 5% CO_2_ at 37˚C for two hours. The infected cells were then washed three times using PBS to remove non-adherent bacteria and then lysed using sterile water. The cell lysate was cultured anaerobically for 4-5 days when bacterial CFUs were counted. Adhesion was determined the number of CFUs recovered from lysates expressed as a percentage of total CFUs recovered from the inoculum. To examine invasion, H357 and H376 cells (5x10^4^) were seeded in 8-well µ-slides (Ibidi, Munich, Germany) to obtain a confluency of 60-70%. *F. nucleatum* suspensions were added (MOI 10:1) for four hours under controlled conditions of 5% CO_2_ and 37˚C. The cells were then treated with 1 mg/ml Hoescht stain, 5 µl of 1 mg/ml propidium iodide (PI) to stain the bacteria and 1 µl of 1 mg/ml fluorescein to stain the extracellular space. Slides were examined using a Leica SP8 Scanning Confocal microscope. 3-D images of epithelial cell invasion were created and the surface area of intracellular PI stained bacteria was quantified in at least 3 fields of view using the Oxford Bitplane Imaris Software (version 9.2.1). Invasion was expressed as µm^2^ of bacteria per cell. To compare invasion levels between cell lines, data were normalised for differences in typical cell size. We quantified the level of intracellular bacteria in at least three independent infection experiments for each strain in H357 and H376 cells.

### Scratch wound assay

Confluent layers of epithelial cells were grown in 6-well plates (Greiner Bio-One GmbH, Austria) and a clear linear scratch was produced in the centre of the well using a sterile micropipette tip. The cells were then infected with *F. nucleatum* subsp. *polymorphum* (MOI 10:1) and incubated in a 37˚C with 5% CO_2_ for 24 hours. The wound healing was calculated measuring the width of the wound at time 0 and time 24 h using a Zoe fluorescent cell imager (BioRad, CA, USA). Data were recorded from three biological replicate experiments.

### Trans-well cell invasion assay

ECM gel (Sigma-Aldrich) was diluted to a final concentration of 1 mg/ml and stored at -20˚C and thawed overnight at 4˚C prior to use. Millicell cell culture inserts (8.0 µm pore, 12 mm well diameter; Millipore, Sigma-Aldrich) were placed in 24-well plates (Greiner Bio-One GmbH, Austria) and chilled to 4˚C before 40 µl of ECM gel was added into the upper compartment. The plate was immediately placed in a 37˚C incubator with circulating 5% CO_2_ for 2-3 hours for the ECM gel to set. Conditioned media (400 µl) obtained from 24 h *F. nucleatum* infections of epithelial cells were added to the wells, ensuring contact with the base of the inserts. Epithelial cells (1 x 10^5^) in 200 µl serum free DMEM were added to the upper compartment of the inserts and incubated at 37˚C with 5% CO_2_ for 48 h. The inserts were removed and the insert membrane was fixed with 3.7% formalin for 10 minutes, followed by staining with 1% crystal violet in 2% ethanol for 20 minutes. The cells were washed thoroughly to remove excess stain and successfully migrated cells were counted using a light microscope. All experiments were performed with three biological replicates.

### HUVEC tube formation assay

Tube formation assays were carried out as described by Fromm et al (Fromm et al., 2019). HUVEC cell culture medium was used to prepare bacterial suspensions for infections of H357 and H376 cells (MOI 10:1) and after 24 h this conditioned medium (CM) was recovered. Matrigel matrix (Corning, USA) was defrosted just before the commencement of the assay and chilled on ice. 50 μl of Matrigel was pipetted into each of the experimental wells of the 96-wells plate and incubated at 37˚C and 5% CO2 for 30 min to solidify the gel matrix. Once set, 8 x 10^4^ HUVEC cells in 80 μl were added to each well along with 120 μl CM. Positive control wells contained 50 ng/ml recombinant human VEGF-A (rVEGF-A; R&D Systems) in HUVEC cell culture media. Experiments were incubated at 37˚C and 5% CO2 for 12 hours to promote tube formation. Tube formation was quantified at 200x magnification by counting the number of capillary-like branches formed. Capillary counts from three fields per well were considered and an average tube formation of the three fields was reported.

### RNA sequencing

H357 and H376 cells were infected with *F. nucleatum* 23726 (MOI 10:1) for 3 h and then RNA was extracted using the RNeasy minikit (Qiagen). Strand-specific, polyA mRNA libraries were sequenced using the Illumina NovaSeq 6000, generating paired end 150 bp reads, by Novogene (Cambridge, UK). Samples were sequenced in triplicate with a minimum of 45 m reads (>6.0 Gb data) per sample with Q30 >90%. At least 94% of reads mapped to the human genome (*Homo sapiens* GRCh38/hg38). Biological replicate samples exhibited Pearson correlation R^2^ values >0.95. DESeq2 was used for analysis of differential expression between infected and uninfected samples (3 biological replicates each) and these gene sets were subjected to Gene Ontology (GO) enrichment analysis to identify processes induced or repressed by *F. nucleatum* infection (Love et al., 2014).

## Results

### Phenotype of malignant oral cells

**Figure 1:**
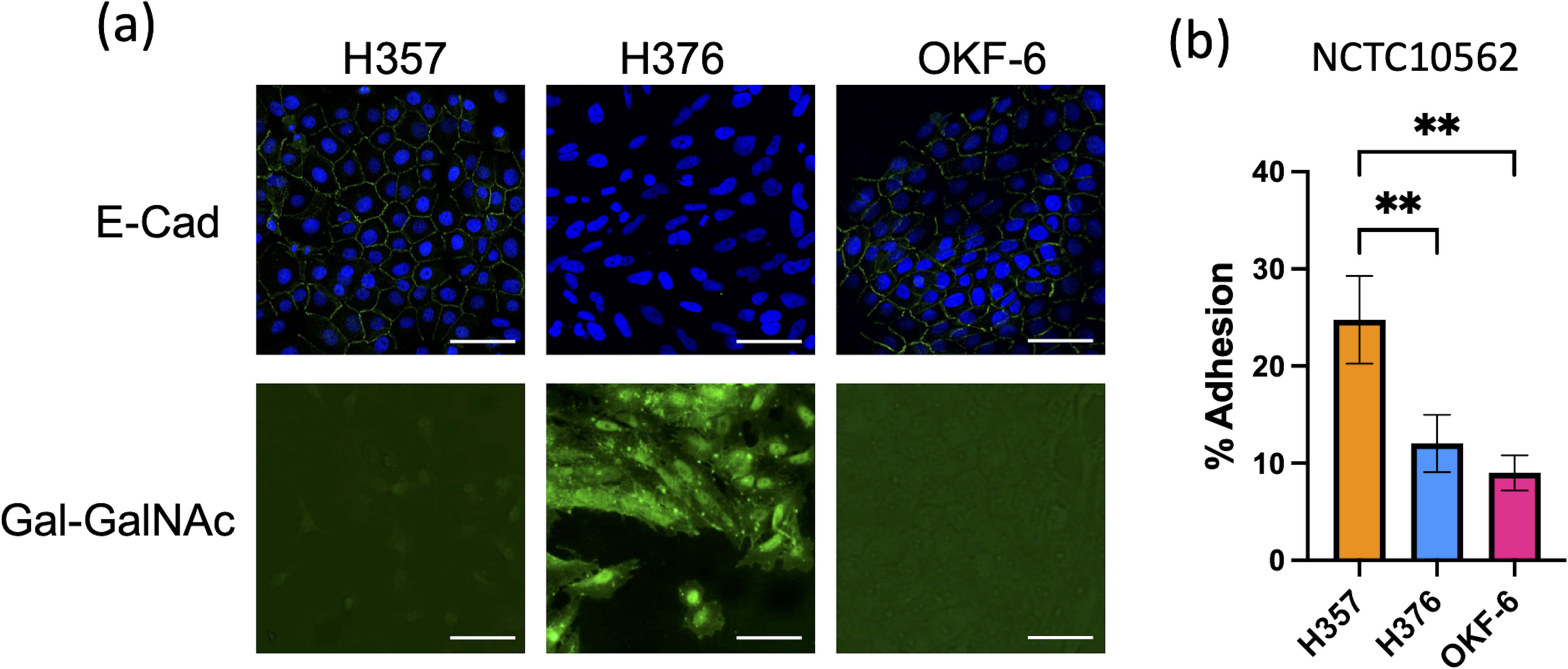
Characterisation of oral keratinocyte cell lines. **(A)** Top row shows immuno-staining of H357, H376 and TERT-1/OKF6 cells for E-cadherin expression using anti-E-cadherin antibody (staining green fluorescent junctions) and DAPI stained nuclei in blue. Bottom row shows staining of H357, H376 and TERT-1/OKF6 cells with FITC labelled peanut agglutinin. The scale bar represents 75 µm in length. **(B)** Adhesion of *F. nucleatum* subsp *polymorphum* strain NCTC10562 to H357, H376 and TERT-1/OKF6 cells (** = ANOVA p <0.001).

We wished to examine the interaction of *F. nucleatum* with two malignant oral keratinocyte cell lines, H357 and H376 (Connolly et al., 2016). We first examined the phenotypes of these cells, initially characterising expression of the cell adhesion molecule E-cadherin. We included the telomerase immortalised oral cell line TERT-1/OKF6 as a control for E-cadherin expression and as expected, these cells stained positively for E-cadherin (**Figure 1**). H357 cells, which originated from a stage 1 tumor, also exhibited E-cadherin expression at cell-cell junctions whereas the H376 cells, originating from a stage 3 tumor, were negative (**Figure 1**). Conversely, the E-cadherin negative H376 cells stained extensively with peanut agglutinin lectin, recognising Gal-GalNAc residues, whereas the E-cadherin positive H357 cells and TERT-1/OKF6 cells exhibited weaker expression of Gal-GalNAc (**Figure 1**). We next examined whether *F. nucleatum* exhibited adhesion to these cell lines using the type strain *F. nucleatum* subsp. *polymorphum* NCTC10562 (equivalent to ATCC10953). This strain exhibited significantly greater adhesion to the H357 cells (~25% adhesion) compared to the H376 cells (~11% adhesion) and the OKF6 cell line (**Figure 1B**).

### Adhesion to oral keratinocytes

**Figure 2:**
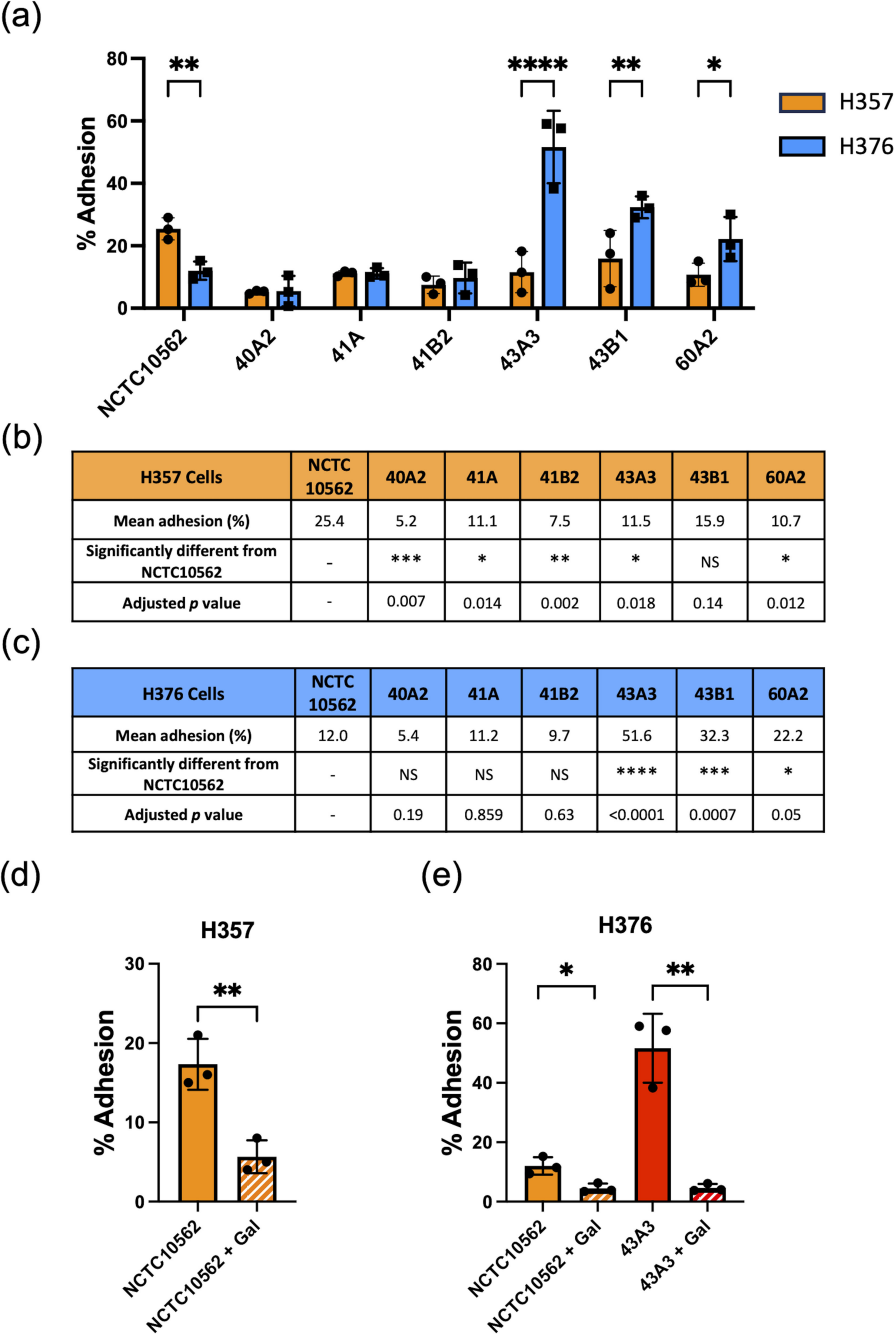
Adhesion of *F. nucleatum* subsp. *polymorphum* clinical strains to oral keratinocytes. **(A)** Adhesion of *F. nucleatum* subsp*. polymorphum* strains to E-cadherin positive H357 and E-cadherin negative H376 cells. Significant differences between cell lines indicate by asterisks (adjusted *p*= *<0.05, **<0.01, *** < 0.001, **** < 0.0001). **(B, C)** Comparisons of adhesion of NCTC10562 and the indicated clinical isolates of *F*. *nucleatum* subsp*. polymorphum* to **(B)** H357 and **(C)** H376 cells using ANOVA with Dunnett’s multiple comparison test. **(D)** Impact of galactose (60 mM) on the adhesion of NCTC10562 to H357 cells (t-test *p*= **<0.01). **(E)** Impact of galactose (60 mM) on the adhesion of strains NCTC10562 and 43A3 to H376 cells (t-test *p*= *<0.05, **<0.01).

We have previously shown that *F. nucleatum* subsp. *polymorphum* is the most frequently cultured subspecies of *F. nucleatum* recovered from oral mucosal surfaces (both normal and potentially malignant oral leukoplakia [OLK]) (Crowley et al., 2024). In the current study, we wished to determine if adhesion of *F. nucleatum* subsp. *polymorphum* varied with bacterial strain and keratinocyte phenotype. For this, we examined adhesion to H357 and H376 cells by a panel of *F. nucleatum* subsp. *polymorphum* isolates recovered from OLK or normal mucosa (**Table 1**). As reported by Crowley et al. (Crowley et al., 2024) genome sequencing of these isolates revealed variation in copy number of encoded FadA-like and Type V autotransporter adhesins (**Table 1**). As noted in **Figure 1**, the type strain NCTC10562 exhibited greater adherence to the H357 cells relative to H376 cells. Two different patterns of adhesion were observed in the clinical strains; 40A2, 41A and 41B2 exhibited similar adhesion to both cell lines, whereas isolates 43A3, 43B1 and 60A2 exhibited greater adhesion to the stage 3 tumor-derived H376 cells (**Figure 2A**). Comparisons between the isolates showed that the adhesion of NCTC10562 to stage 1 tumor-derived H357 cells was greater than any of the clinical isolates tested (**Figure 2B**) whereas its adhesion to the H376 cells was significantly lower compared to isolates 43A3, 43B1 and 60A2 (**Figure 2C**).

In order to determine if adhesion involved lectin interactions with galactose residues on oral keratinocytes, we pre-treated the bacterial strains with 60 mM galactose for 30 minutes before performing the assays. We found that 60 mM galactose pre-treatment significantly reduced adhesion of NCTC10562 to both cell lines (**Figures 2D, E**). We also examined if lectin interactions were involved in the high level adhesion exhibited by strain 43A3 to the H376 cell line and found that adhesion of 43A3 was very significantly reduced by galactose (**Figure 2E**).

### Invasion of oral keratinocytes

**Figure 3:**
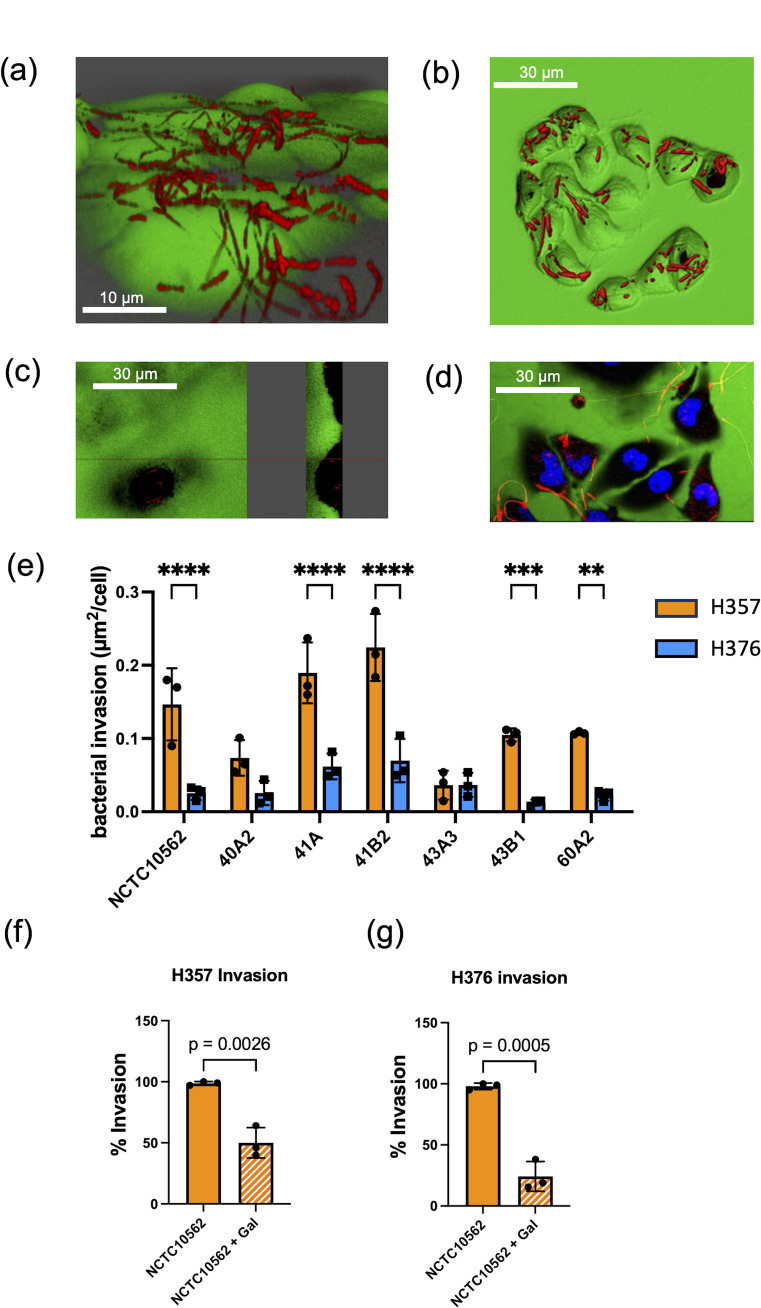
Invasion of oral keratinocytes by *F. nucleatum* subsp*. polymorphum*. **(A)** Propidium iodide (PI) stained cells of *F. nucleatum* subsp. *polymorphum* NCTC10562 interacting with H357 oral keratinocytes. **(B)** 3D confocal image of H376 oral keratinocytes with internalised cells of *F*. *nucleatum* subsp. *polymorphum* NCTC10562 stained with PI. **(C)** Saggital-section of H357 epithelial cells showing internalised bacteria of strain NCTC10562. **(D)** Confocal Z stack image of invasion assay of H357 cells (nuclei stained using Hoescht) being invaded by strain NCTC10562. **(E)** Bacterial invasion of H357 and H376 oral keratinocytes quantified using Imaris software as µm^2^ of bacteria per cell. Significant differences in the invasion of H357 and H376 cells indicated by asterisks (ANOVA adjusted p = ** < 0.01, *** <0.001, **** <0.0001). **(F)** Impact of galactose (60 mM) on the invasion of H357 cells by strain NCTC10562 with t-test *p* value. **(G)** Impact of galactose (60 mM) the invasion of H376 cells by strain NCTC10562 with t-test *p* value.

In order to determine if *F. nucleatum* subsp. *polymorphum* could invade these cell lines, we carried out confocal microscopy. Staining of *F. nucleatum* subsp. *polymorphum* with propidium iodide (PI) clearly showed adhesion to and penetration of the plasma membrane by Fusobacteria (**Figure 3A**) and also showed the presence bacteria in the intracellular compartment (**Figure 3B**), which was confirmed in sagittal sections of infected cells (**Figure 3C**) and z-stacked images (**Figure 3D**). Quantification of intracellular bacterial fluorescence facilitated measurement of the surface area of bacteria per cell (µm^2^/cell). With the exception of strain 43A3, all *F. nucleatum* isolates examined exhibited significantly greater invasion of H357 cells compared to H376 cells (**Figure 3E**). Some variation was observed in the ability of strains to invade H357 cells, with strain 41B2 exhibiting significantly greater invasion than NCTC10562, and strains 40A2 and 43A3 exhibiting significantly weaker invasion (**Supplementary Figure S1**). Similarly strain variation was observed in relation to invasion of H376 cells with strains 41A and 41B2 both showing significantly greater invasion than NCTC10562 (**Supplementary Figure S1**).

We also investigated if invasion was dependent on lectin interactions with galactose residues on oral keratinocytes. We pre-treated strain NCTC10562 with 60 mM galactose for 30 minutes before performing the assays. We observed that galactose pre-treatment significantly reduced invasion both H357 and H376 cells by strain NCTC10562 (**Figures 3F, G**).

### Analysis of cellular responses to infection

**Figure 4:**
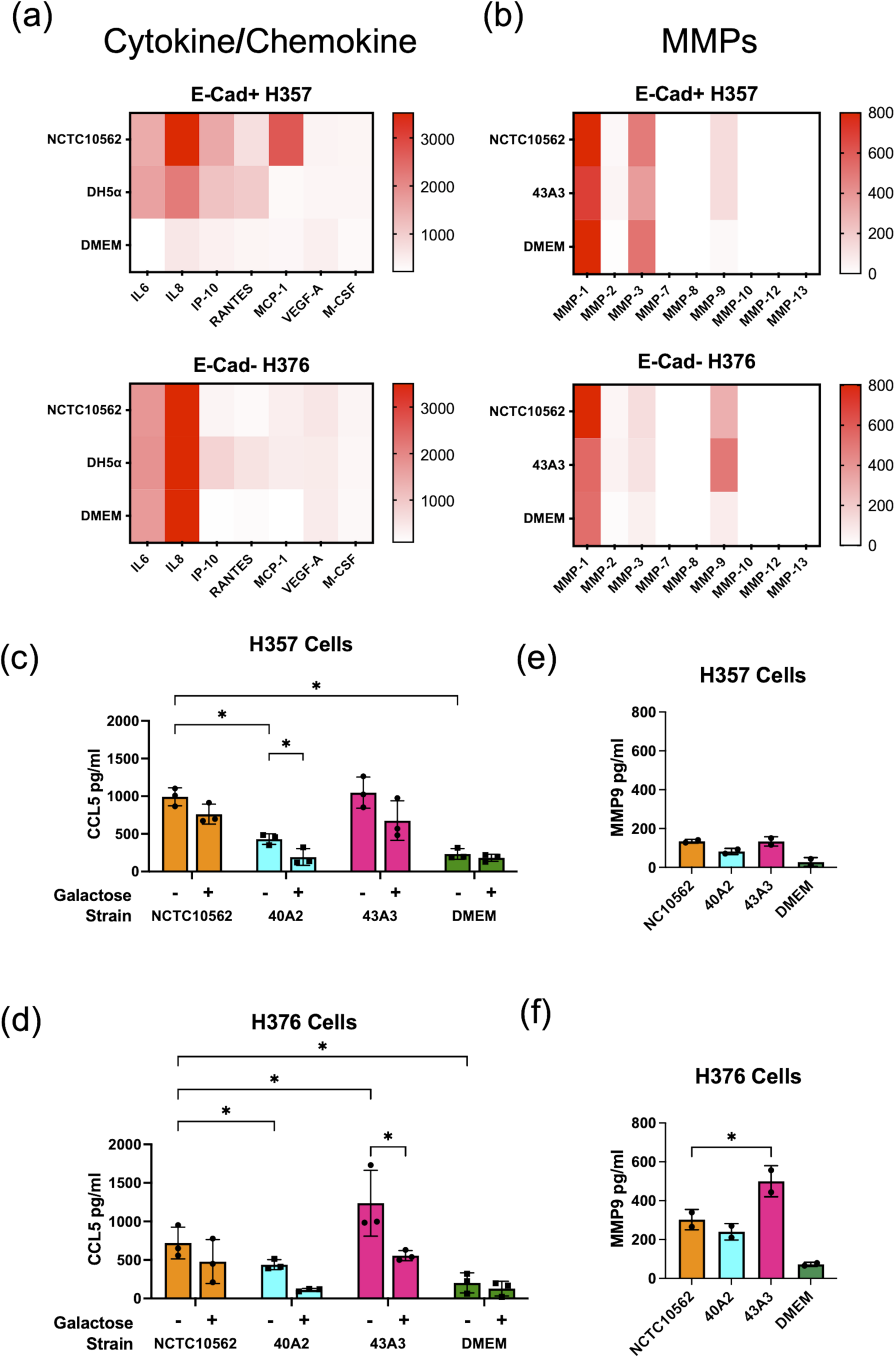
Response of H357 and H376 oral keratinocytes to *F. nucleatum* infection. **(A)** Expression of selected cytokines and chemokines (pg/ml) detected in a multiplex assay (Eve Technologies, Canada) in cell culture media (DMEM) recovered from H357 and H376 cells infected with *F. nucleatum* subsp. *polymorphum* NCTC10562 (MOI 10:1) or *E. coli* DH5α (MOI 50:1). **(B)** Expression of MMPs (pg/ml) detected in a multiplex assay (Eve Technologies, Canada) in cell culture media (DMEM) recovered from H357 and H376 cells infected with *F. nucleatum* subsp. *polymorphum* NCTC10562 or 43A3. **(C)** ELISA of CCL5 expression in H357 cells and **(D)** H376 cells uninfected (DMEM only) and infected with the indicated strains of *F. nucleatum* subsp. *polymorphum* in the presence or absence of galactose. Asterisk indicates significant differences identified in 2-way ANOVA corrected for multiple comparisons (adjusted *p=* *<0.05). **(E)** ELISA results of MMP9 expression in H357 and **(F)** H376 cells uninfected (DMEM only) and infected with the indicated strains of *F. nucleatum* subsp. *polymorphum*. Asterisk indicates significant difference from NCTC10562 in ANOVA with Dunnett’s test for multiple (adjusted *p=* *<0.05).

We next analysed the cytokine response of the H357 and H376 cells to infection with NCTC10652 and clinical strains of *F. nucleatum* subsp. *polymorphum*. In the absence of infecting bacteria, both cell-lines secreted baseline levels of IL-8, which were greatest in the E-cadherin negative H376 cells (**Figure 4A**). Infection with NCTC10562 was seen to induce a variety of inflammatory cytokines and chemokines including IP-10, RANTES/CCL5 and MCP-1/CCL2 (**Figure 4A**). These responses were also induced by *E. coli* DH5α (MOI 50:1), indicating that this may be a general response to Gram-negative bacterial infection. As RANTES/CCL5 has been previously linked to MMP9 expression and tumor cell migration, we confirmed induction of CCL5 expression using ELISA. CCL5 was induced in both cell lines by NCTC10562 and the clinical strains 40A2 and 43A3. Differences in induction could be discerned, with the low adhesion isolate 40A2 inducing significantly less secretion than NCTC10562 in H357 and H376 cells and the high adhesion isolate 43A3 inducing significantly higher secretion compared to NCTC10562 in H376 cells. In this case, inhibition of adhesion of 43A3 to H376 cells with galactose (60mM) could significantly reduce secretion of CCL5 (**Figure 4D**).

Multiplex analysis of MMP expression also indicated that *F. nucleatum* infection induces secretion of MMP9 in both cell lines. As MMP9 has been linked to tumor cell invasion and migration we examined MMP9 expression using ELISA (**Figures 4E, F**). H376 cells expressed higher levels of MMP9 following *F. nucleatum* infection and these were significantly higher in cells infected with the high adhesion isolate 43A3 (**Figures 4E, F**).

### Transcriptional responses to *F. nucleatum*

**Figure 5:**
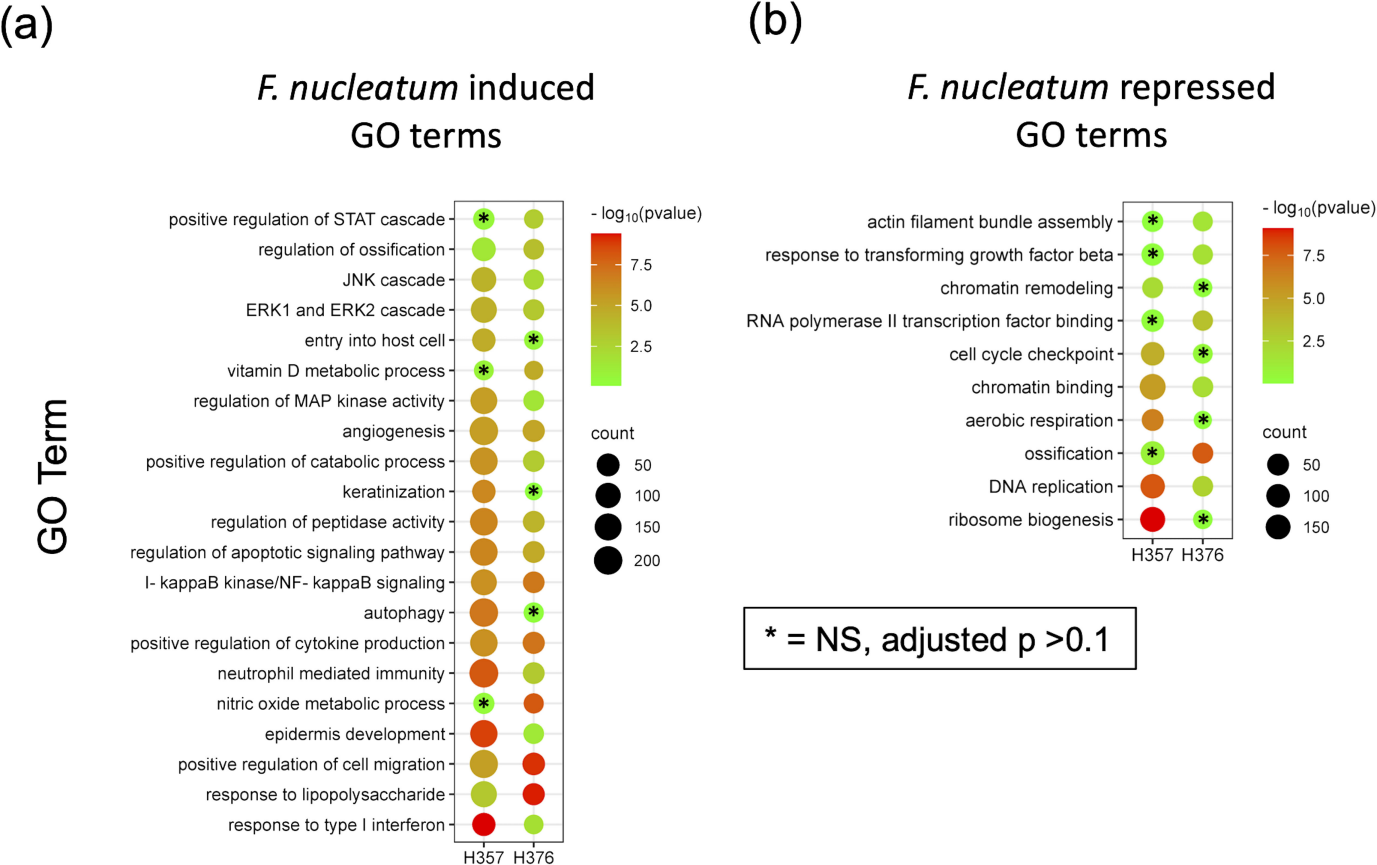
RNA-Seq analysis of the response of H357 and H376 oral keratinocytes to infection with *F. nucleatum* 23726. Oral keratinocytes were infected with *F. nucleatum* for 3 h at an MOI of 10:1. Plot shows the results of Gene Ontology (GO) term enrichment analysis in the **(A)** upregulated and **(B)** down regulated gene sets in both cell lines. Adjusted *p* values were generated using the Benjamini-Hochberg FDR method. The most significant non-redundant GO terms were selected for display. Cell lines with adjusted p values >0.1 are indicated by asterisks. A full list can be seen in **Supplementary Tables S3**, **S4**.

In order to support the findings of the cytokine data, we analysed transcriptomic data from H357 and H376 oral keratinocytes infected with *F. nucleatum* in order to determine if transcriptional responses related to motility and invasion are activated. Although this analysis was performed with *F. nucleatum* subsp. *nucleatum* ATCC23726, the data are likely to reflect responses activated by *F. nucleatum* in general. Infection of both cell lines activated transcriptional responses, with the H357 cell line exhibiting a greater number of significantly regulated genes compared to uninfected cells (1423 up and 669 down genes; *p <*0.05) compared to the H376 cells (543 up and 363 down genes, *p <*0.05) (**Supplementary Figure S2**; **Supplementary Tables S1**, **S2**). As expected, both cell lines activated a response to Gram-negative infection, with significant enrichments in expression of genes within the GO terms “response to lipopolysaccharide”, “positive regulation of cytokine activity” and “NF-κB signalling” (**Figure 5**; **Supplementary Tables S3**, **S4**). Both cell lines also activated angiogenic responses and responses involved in cellular mobility and migration (**Figure 5**). Uniquely, the H357 cell line underwent a transcriptional response that suggests a major shift in metabolism, involving reduced aerobic respiration, ribosome biogenesis and increased catabolism and autophagy (**Figure 5**). The H357 cells also activated responses involved in keratinization and epidermis development (**Figure 5**). Uniquely, the H357 cells also activated a response termed “entry into host cell” including genes that may be induced upon pathogen entry. Fewer specific responses were identified in the E-cadherin negative H376 cell line, however some responses such as the response to lipopolysaccharide and cell migration categories exhibited more highly significant adjusted *p* values due to the smaller size of the total gene set.

### *F. nucleatum* subsp*. polymorphum* induces migration and invasion by H357 and H376 keratinocytes

**Figure 6:**
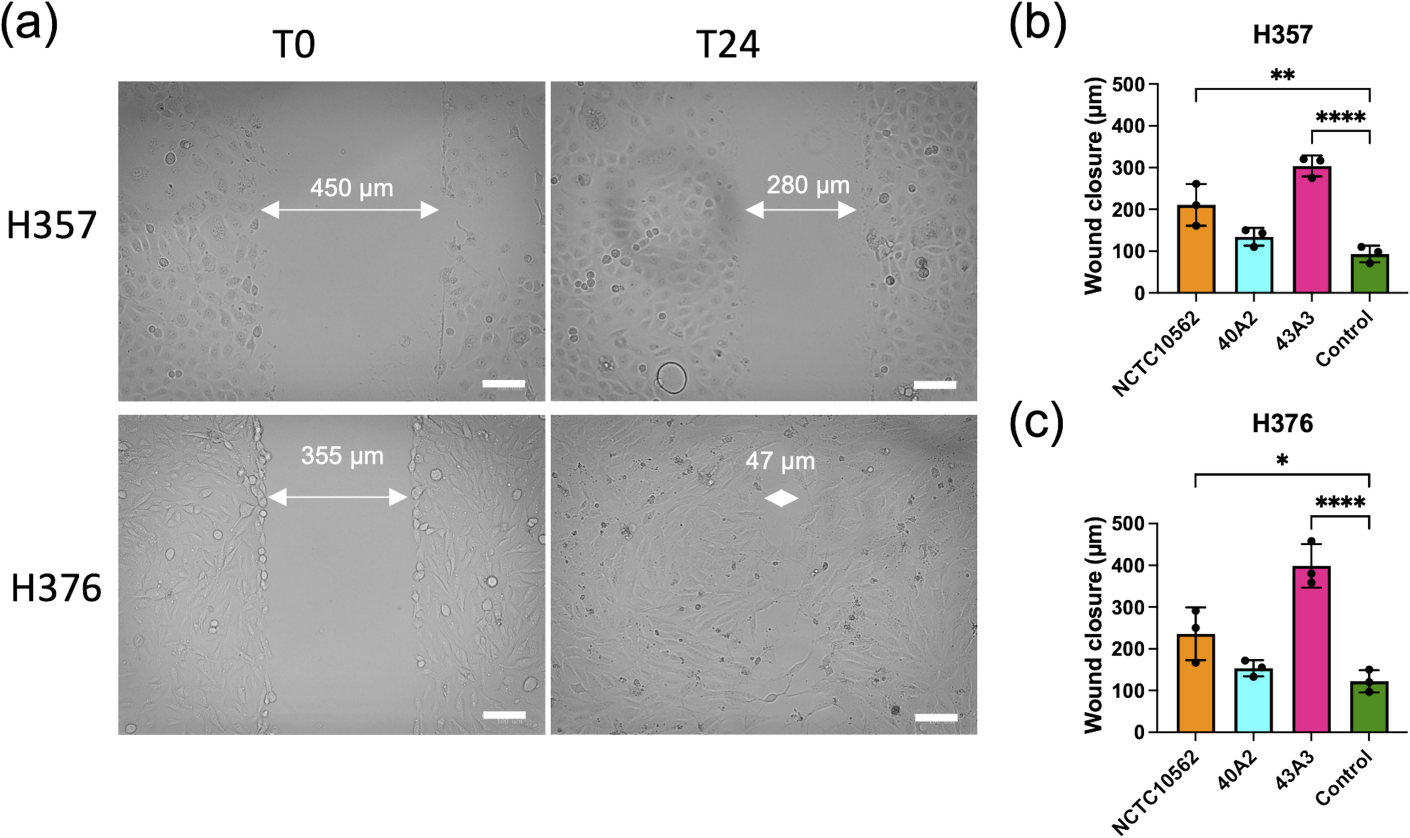
Scratch wound assay. **(A)** Representative images of scratch wounds in H357 cells (top row) and H376 cells (bottom row). Left panel shows cell at time 0 and right panel after 24 h infections with strain NCTC10562. Arrows indicate scratch wound width (µM). Scale bar=100 μm. **(B)** Wound closure measurements in H357 cells and **(C)** H376 cells following incubation with DMEM (control) or infections with the indicated strains. Cell migration was assessed by calculating the amount of wound closure after 24 h infection. Treatments significantly different from the control (DMEM) are indicated by asterisks (ANOVA adjusted p = * <0.05, ** < 0.01, **** <0.0001).

**Figure 7:**
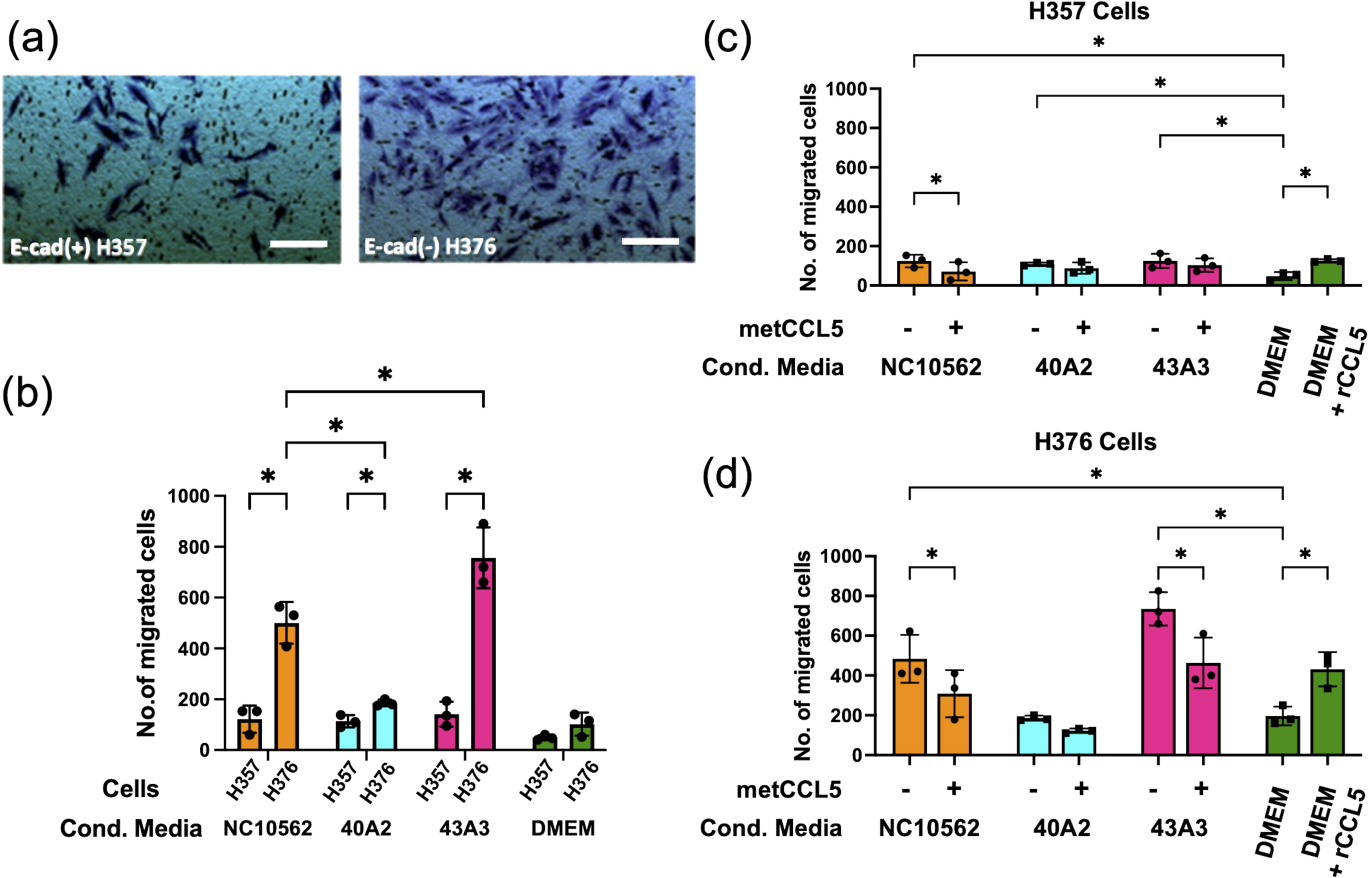
Analysis of invasive phenotypes in H357 and H376 cells in response to CM from cells infected with *F. nucleatum* subsp. *polymorphum.* **(A)** Representative image showing stained oral keratinocytes following migration across the matrix coated filter. The white lines represent scale bar=100 μm **(B)** Comparison of migration of H357 and H376 keratinocytes following treatment with CM from the indicated strains. Asterisks indicate results of 2-way ANOVA (adjusted *p*= *<0.05). **(C)** Analysis of H357 and **(D)** H376 cell invasion following treatment with CM from the indicated strains with and without pre-treatment with the inhibitor metCCL5. Asterisks indicates CM treatments significantly different from DMEM and significant reductions in invasion following metCCL5 treatment (2-way ANOVA adjusted *p*= *<0.05).

To assess the impact of *F. nucleatum* subsp. *polymorphum* infection on the motility and invasiveness of oral keratinocytes, we assessed the responses of H357 and H376 cells using a scratch wound assay and trans-well invasion assay. For the scratch wound assay (**Figure 6A**), keratinocytes were infected by *F. nucleatum* subsp. *polymorphum* (MOI 10:1) for 24 hours. Infection of keratinocytes (both H357 and H376) by strains NCTC10562 or 43A3 resulted in significantly greater migration in a scratch wound assay (**Figures 6B, C**). Next, we examined keratinocyte invasiveness in response to CM by measuring keratinocyte migration across matrix coated filters (**Figure 7A**). In general, we observed that H376 cells exhibited greater migration in response to CM compared to H357 cells (**Figure 7B**). However the response of H376 cells varied between strains with significantly greater invasion of H376 cells observed with CM from strain 43A3 and significantly less with CM from strain 40A2 compared to strain NCTC10562. Recombinant CCL5 could also enhance migration of both cell lines (**Figures 7C, D**). Compared to DMEM alone, CM from strains NCTC10562 and 43A3 induced the most significant increases in migration (**Figures 7C, D**). Next, we examined whether blockage of CCL5 signalling using the inhibitor metCCL5 (3 ng/ml pre-treatment for 30 min) could reduce the migration response to CM. We observed a significant reduction in migration of both H357 and H376 cells in response to NCTC10562 CM following metCCL5 treatment (**Figures 7C, D**). We also observed a significant reduction in the migration of H376 cells in response to CM from 43A3 following metCCL5 treatment (**Figure 7D**).

### *F. nucleatum* subsp*. polymorphum* induces angiogenic responses in H357 and H376 keratinocytes

**Figure 8:**
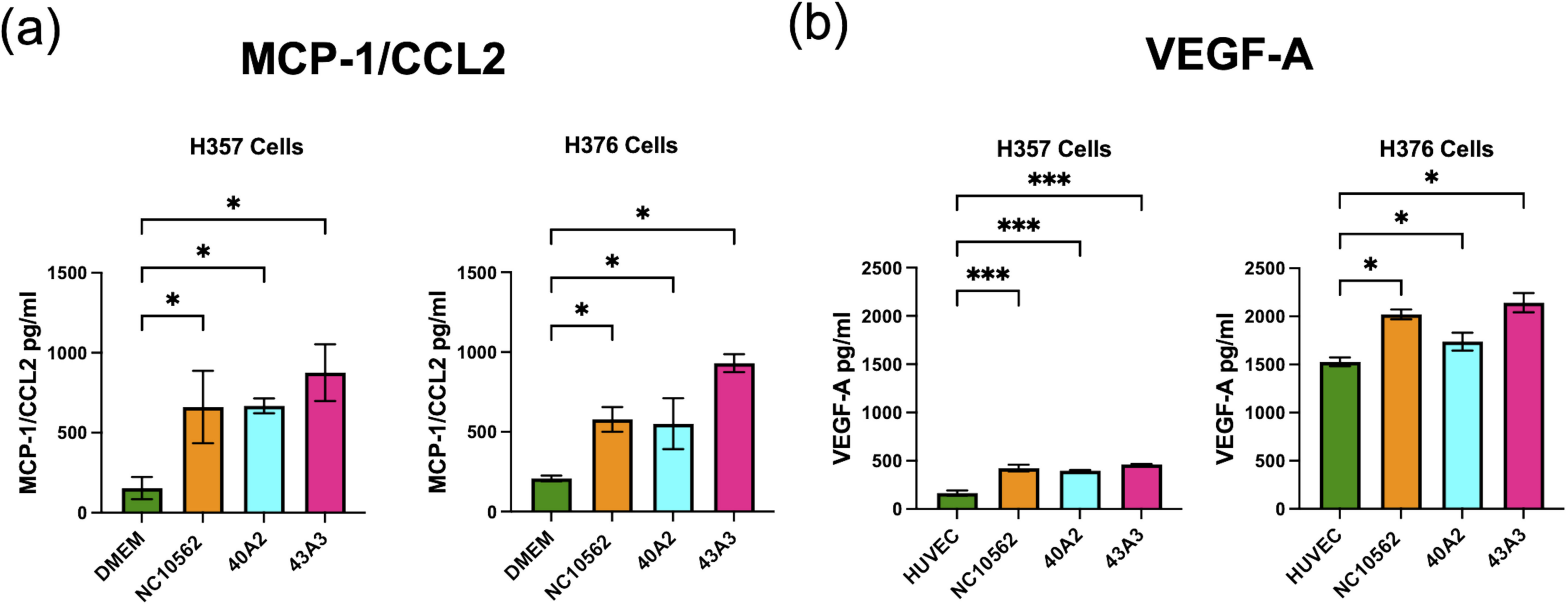
Analysis of secretion of **(A)** MCP-1/CCL2 and **(B)** VEGF-A by H357 and H376 oral keratinocytes following *F. nucleatum* infection. ELISA quantification was carried out on culture medium (DMEM or HUVEC) following 24 h infection with the indicated strains of *F. nucleatum* subsp, *polymorphum* (MOI 10:1). Significant differences from uninfected cells was calculated using ANOVA with Dunnett’s test for multiple comparisons (adjusted *p* = *<0.05, ***<0.001).

Based on the pro-angiogenic transcriptional responses (**Figure 4**) and the observed induction of CCL2/MCP-1 in our multiplex assays (**Figure 5**), we investigated whether *F. nucleatum* subsp. *polymorphum* infection could induce the angiogenic factor VEGF-A or angiogenic responses in HUVEC cells. CCL2/MCP-1 is known to induce VEGF-A expression in malignant cells via stimulation of its cognate receptor CCR2. Using ELISA we found that clinical strains 40A2, 43A3 and the type strain NCTC10562 could induce significantly increased secretion of CCL2 in both H357 and H376 cells (**Figure 8A**). We next investigated the impact of *F. nucleatum* subsp. *polymorphum* infection on VEGF-A secretion. Without *F. nucleatum* stimulation, the H376 cells were found to secrete significantly higher baseline levels of VEGF-A compared to H357 cells (t-test *p <*0.001). However, in the case of both cell lines, exposure to *F. nucleatum* subsp. *polymorphum* increased VEGF-A secretion (**Figure 8B**).

**Figure 9:**
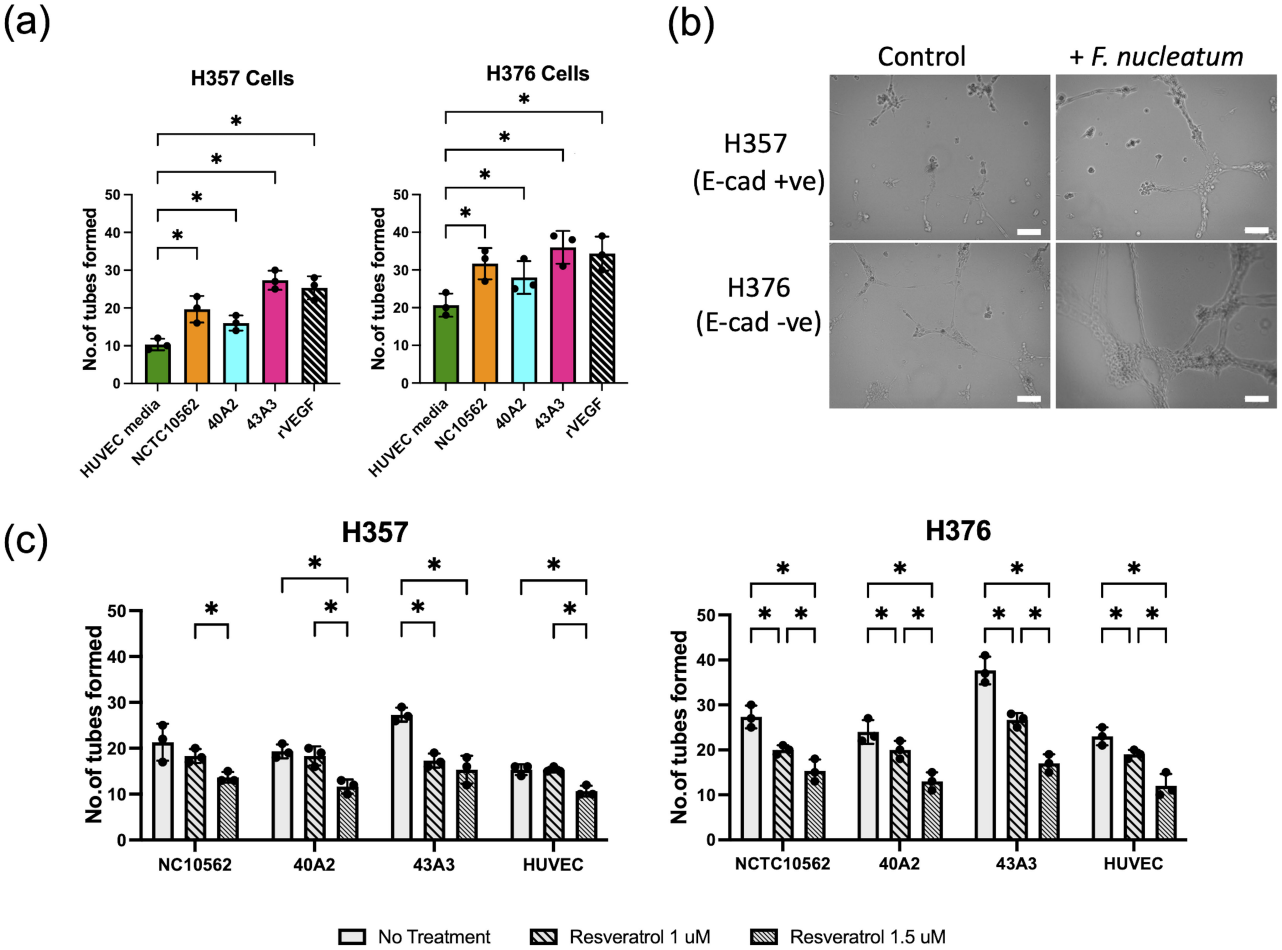
Analysis tube formation by HUVEC cells. **(A)** Quantification of tube formation in HUVEC cells exposed to conditioned HUVEC medium from H357 and H376 oral keratinocytes following *F. nucleatum* infection (24 h infection, MOI 10:1). Recombinant VEGF-A (rVEGF; 50 ng/ml) is included as a positive control. Significant differences from uninfected control (HUVEC medium) was calculated using ANOVA with Dunnett’s test for multiple comparisons (adjusted *p* = *<0.05). **(B)** Representative images of HUVEC cells after 12 h exposure to conditioned HUVEC medium obtained from H357 cells (top row) or H376 cells (bottom row) that were uninfected (control) or infected with *F. nucleatum* strain 43A3 (+ *F. nucleatum*). The white lines represent scale bar=100 μm. **(C)** Inhibition of HUVEC tube formation by resveratrol (1 μM and 1.5 μM). Significant differences between treatments were identified using 2-way ANOVA (*q* = *<0.05).

We next examined if CM from infected cells could enhance angiogenesis in HUVEC cells by measuring tube formation. Supplementation of sterile HUVEC medium with recombinant human VEGF-A (rVEGF) resulted in a significant increase in tube formation (**Figure 9A**). Tube formation was also induced by conditioned HUVEC media recovered from both uninfected cell lines, with medium from the high VEGF-A secreting H376 cells inducing greater tube formation than H357 cells (**Figure 9A**). Next we examined if exposure to conditioned HUVEC media produced following infection of the cells lines with *F. nucleatum* subsp. *polymorphum* could enhance tube formation to a similar extent. In the case of both H357 and H376 cells, CM from infections with all three *F. nucleatum* subsp. *polymorphum* strains, NCTC10562, 40A2 and 43A3, resulted in significantly increased tube formation compared to HUVEC medium from uninfected cells (**Figures 9A, B**). In order to confirm whether these phenotypes were VEGF-A specific, we treated cells with resveratrol, which has been shown to inhibit VEGF receptor 2 phosphorylation and signalling. Exposure of HUVEC cells to 1.5 µM resveratrol could reduce tube formation in all preparations of conditioned media (**Figure 9C**).

## Discussion

Our previous studies identified *F. nucleatum* subsp. *polymorphum* as the most common subspecies recovered from healthy and diseased oral mucosa (including OLK and OSCC) (Crowley et al., 2024). These data concur with Krieger et al. (Krieger et al., 2024) who show that subsp. *polymorphum* is the *F. nucleatum* subspecies with the highest abundance in the oral microbiome (Krieger et al., 2024). One of the most surprising aspects of our genetic analysis of these strains was the level of heterogeneity in the accessory genome, especially in copy number of Fap2-like autotransporter adhesins and copy number of FadA-like adhesins. The overarching goal of the current study was to determine if this genetic heterogeneity manifested in phenotypic differences in host-cell interaction. The clinical isolates selected were previously shown to differ in adherence to oral keratinocytes and to vary in their repertoire of adhesin genes (Crowley et al., 2024). In addition, we evaluated the interaction with two different malignant cell lines which differed in origin in terms of tumor stage (stage 1 v stage 3). The H376 cells had lost expression of E-cadherin and showed increased expression of Gal-GalNAc, properties typical of a more advanced tumor, whereas the H357 cells from a stage 1 tumor still expressed E-cadherin and were morphologically more similar to the immortalized OKF-6 cells (Fardini et al., 2011; Coppenhagen-Glazer et al., 2015).

We could detect considerable variation in adhesion to these oral keratinocytes, with the type strain NCTC10562 exhibiting a strong preference for adhesion to the H357 cells, whereas 3 of the 6 clinical isolates examined exhibited a preference to the H376 cells. Galactose was shown to inhibit the interaction of bacteria with both cell lines, indicating the importance of lectin binding in these interactions. Strain 43A3, which exhibited extremely high levels of adhesion to the Gal-GalNAc expressing H376 cell line was very significantly inhibited by galactose (~85%). These data also suggest that as epithelial dysplasia advances and Gal-GalNAc expression increases (Abdalla et al., 2017) strains of *F. nucleatum* that adhere strongly to Gal-GalNAc residues may be selected. The identification of clinical isolates here exhibiting high adhesion to Gal-GalNAc expressing cells could be the result of *in vivo* selection for dysplasia adapted genotypes. In the absence of efficient gene deletion tools for *F. nucleatum* subsp. *polymorphum* it is impossible to dissect the adhesin-receptor interactions at play. However what these data illustrate is the heterogeneity in adhesive phenotypes among clinical isolates of subspecies *polymorphum* that possess different repertoires of adhesins. *F. nucleatum* is highly recombinogenic which may facilitate the generation of variants that can adhere to different surfaces (Mira et al., 2004).

Keratinocyte invasion by *F. nucleatum* was greatest in the H357 cell line. We observed lower levels of invasion of H376 cells, even by isolates which exhibited strong adherence to these cells. The H357 cell line was shown to express E-cadherin, which could be involved in receptor mediated internalization via the FadA adhesin on the surface of *F. nucleatum* (Ikegami et al., 2009). All of the isolates under investigation possess FadA and FadA-related adhesins (FadA2, FadA3) which may mediate these interactions. Although the role of E-cadherin in this phenotype may require knock-down experiments to confirm, our data show that invasion of keratinocytes by clinical strains of *F. nucleatum* is highly cell-line dependent and is likely influenced by the expression of different receptors on the epithelial cells. H357 cells, which permitted higher levels of bacterial internalization, also exhibited a stronger transcriptional response to *F. nucleatum*. Specific responses of the H357 cells included a keratinization response, which may be a defensive response to invasion, and a metabolic response that included induction of autophagy, a process previously linked with infection induced chemoresistance in tumor cells (Yu et al., 2017). Both cell lines exhibited induction of transcriptional responses linked to cellular motility and angiogenesis, processes strongly associated with tumor development and metastases (Claffey and Robinson, 1996; Lien et al., 2020; Ralli et al., 2020). Analysis of secreted factors induced following *F. nucleatum* infection supported this association with significant induction of the chemokine CCL5/RANTES and MMP-9 detected in conditioned medium. CCL5/RANTES is a known inducer of MMP9 expression which has been shown to facilitate cellular invasion (Woodhouse et al., 1997; Chuang et al., 2009). Lectin interactions have previously been shown to play a role in chemokine induction by *F. nucleatum* and our data show that disruption of adhesion with galactose can partly attenuate CCL5 induction (Casasanta et al., 2020). We also observed that the highly adherent isolate 43A3 induced higher responses compared to the low adhesion isolate 40A2. However, although lectins such as Fap2 may play significant roles in mediating host cell interaction, the finding that a high MOI infection with *E. coli* (MOI 50:1) could stimulate a somewhat similar cytokine response suggests that this may be a general response to a Gram-negative infection.

Examination of the response of oral keratinocytes to infection also revealed cell line-specific and bacterial strain-specific responses. The E-cadherin negative H376 cell line generally exhibited greater motility and invasion across ECM gel relative to E-cadherin positive cells in our assays. This may be related to the loss of intercellular adhesion afforded by E-cadherins and the greater basal expression of MMP9 (Chuang et al., 2009; Mohamet et al., 2011). Loss of E-cadherins have been associated with aggressiveness, advanced stage and poor prognosis of cancers (Abdalla et al., 2017). Cellular invasion and migration of H376 cells were significantly increased by strains NCTC10562 and 43A3, both of which were stronger inducers of CCL5/RANTES and MMP9 compared to strain 40A2. The involvement of the CCL5 axis in this phenotype could be inferred from the inhibitory effects of metCCL5, a competitor inhibitor for CCR5 receptors, on cell migration (Chuang et al., 2009).

Our transcriptional and chemokine expression data indicate that *F. nucleatum* induces pro-angiogenic responses, which to our knowledge have not been previously reported. VEGF-A, which is essential for the formation of new blood vessels in the body, including those in growing tumors, is regulated by multiple factors, including oncogenes, pro-inflammatory cytokines and chemokines such as MCP-1/CCL2, WNT1-inducible signaling pathway protein-1 (WISP-1/CCN-4), hormonal modulators and hypoxia (Claffey and Robinson, 1996; Lien et al., 2020). Infection of both cell lines with *F. nucleatum* resulted in induction of the chemokine MCP-1/CCL2 which has been shown to induce VEGF-A in OSCC (Lien et al., 2020). We could also demonstrate induction of VEGF-A following *F. nucleatum* infection in both cell lines and this was reflected in the enhanced capillary-like tube formation induced in HUVEC cells. The tube formations were reduced when an increasing concentration of resveratrol, a selective VEGFR inhibitor was used (Igura et al., 2001). Induction of angiogenesis indicates that *F. nucleatum* may exacerbate tumor growth and metastases by stimulating blood vessel formation required for provision of nutrients and oxygen to the growing tumor.

Further studies using animal models of angiogenesis or chorioallantoic membrane (CAM) assays would be needed to confirm these responses. In addition, a larger panel of *F. nucleatum* isolates would be required to determine of any of these phenotypes are stronger in isolates recovered from severe epithelial dysplasia or OSCC.

## Conclusion

In conclusion, our study has uncovered a high degree of phenotypic variability in *F. nucleatum* subsp. *polymorphum*, the most common *F. nucleatum* subspecies associated with healthy and dysplastic oral mucosa. The variability in adhesin gene complement shows that specific strains of subspecies *polymorphum* may be better adapted to adhere to certain cell types, suggesting that tumor development may select for certain *F. nucleatum* genotypes that can bind to tumor-specific markers such as Gal-GalNAc. Crowley et al. have shown that recombination among adhesin-encoding genes occurs in other subspecies of *F. nucleatum* suggesting that inter-strain variability is not likely to be unique to subspecies *polymorphum.* Host cell phenotype also appears to be an important variable in influencing the outcome of *F. nucleatum*-keratinocyte interactions. The E-cadherin expressing H357 cell line examined here, despite harboring more intracellular bacteria and activating stronger transcriptional responses, was generally less motile and less invasive in response to *F. nucleatum* infection compared to the E-cadherin negative H376 cell line. This could suggest that the detrimental effects *F. nucleatum* infection are most pronounced in higher stage tumors where loss of E-cadherin and EMT phenotypes are more advanced, acting as a driver of metastatic phenotypes.

## Data Availability

The datasets presented in this study can be found in online repositories. The names of the repository/repositories and accession number(s) can be found below: https://www.ncbi.nlm.nih.gov/, PRJNA1071052.

## Supplementary material

Supplementary Figure S1 Comparisons of invasion of NCTC10562 and the indicated clinical isolates of *F. nucleatum* subsp*. polymorphum* to H357 and H376 cells.

Supplementary Figure S2 Volcano plots showing differentially expressed genes (p <0.05) in H357 and H376 cells following infection with *F. nucleatum* 23726, identified using DeSeq2.

Supplementary Table S1 Differentially expressed genes in *F. nucleatum* infected H357 cells compared to uninfected cells identified using DeSeq2 (p <0.05).

Supplementary Table S2 Differentially expressed genes in *F. nucleatum* infected H376 cells compared to uninfected cells identified using DeSeq2 (p <0.05).

Supplementary Table S3 Significant GO term enrichments in *F. nucleatum* infected H357 cells compared to uninfected cells (padj <0.05).

Supplementary Table S4 Significant GO term enrichments in *F. nucleatum* infected H376 cells compared to uninfected cells (padj <0.05).
