# Supplementary material for: Fusobacterium nucleatum induces invasive growth and angiogenic responses in malignant oral keratinocytes that are cell line- and bacterial strain-specific
Source: Front Cell Infect Microbiol. 2024 Sep 2;14:1417946. doi: 10.3389/fcimb.2024.1417946 (PMC11402903; doi:10.3389/fcimb.2024.1417946)
Supplement: Supplementary Figure S1 — Comparisons of invasion of NCTC10562 and the indicated clinical isolates of F. nucleatum subsp. polymorphum to H357 and H376 cells. [file DataSheet1.pdf]

Figure S1

(a)

H357 Cells	NCTC 10562	40A2	41A	41B2	43A3	43B1	60A2
Mean invasion (μm^2)	0.146	0.073	0.189	0.224	0.036	0.105	0.107
Significantly different from NC10562	-	*	NS	*	**	NS	NS
Adjusted *p* value	-	0.033	0.35	0.022	0.001	0.38	0.44

(b)

H376 Cells	NCTC 10562	40A2	41A	41B2	43A3	43B1	60A2
Mean invasion (μm^2)	0.025	0.025	0.062	0.07	0.036	0.012	0.022
Significantly different from NC10562	-	NS	*	*	NS	NS	NS
Adjusted *p* value	-	0.978	0.046	0.012	0.91	0.86	0.99

Figure S1. Comparisons of invasion of NCTC10562 and the indicated clinical isolates of *F. nucleatum* subsp. *polymorphum* to H357 (a) and H376 (b) cells using ANOVA with Dunnett's test for multiple comparisons.

Figure S2

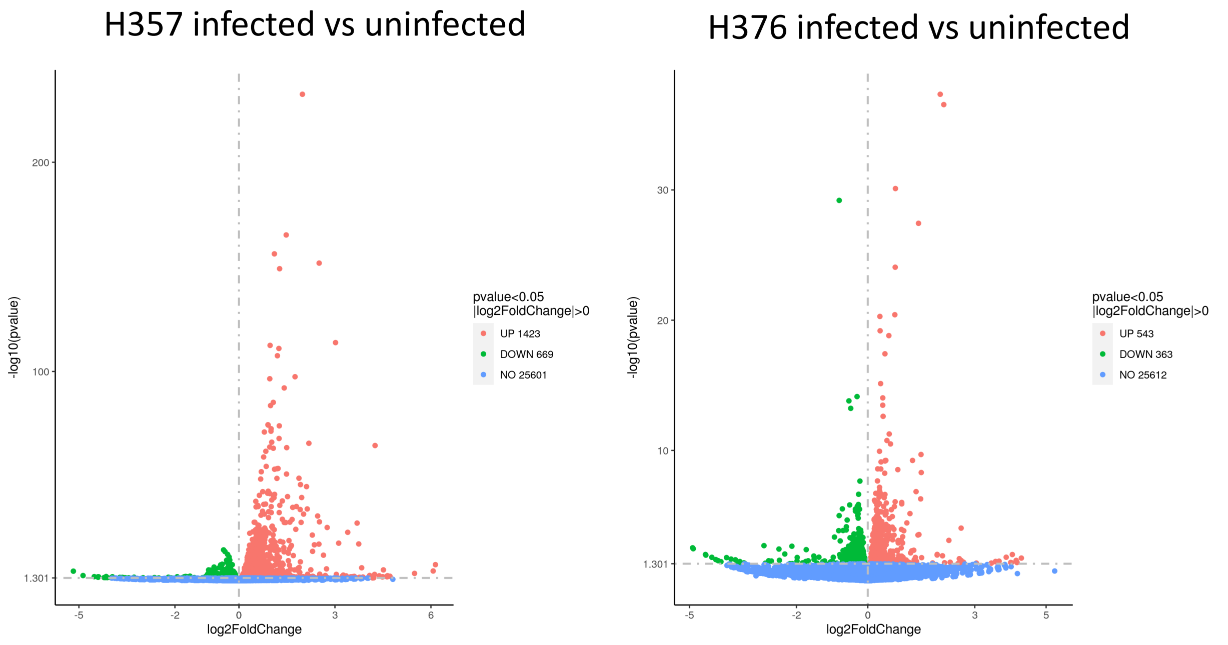

Figure S2. Volcano plots showing differentially expressed genes (p <0.05) in H357 and H376 cells following infection with *F. nucleatum* 23726, identified using DeSeq2.
